# Continuous mowing differentially affects floral defenses in the noxious and invasive weed *Solanum elaeagnifolium* in its native range

**DOI:** 10.1038/s41598-024-58672-w

**Published:** 2024-04-07

**Authors:** Alejandro Vasquez, Alexa Alaniz, Robert Dearth, Rupesh Kariyat

**Affiliations:** 1 Department of Entomology and Plant Pathology, University of Arkansas, Fayetteville, AR 72701 USA; 2 Department of Biology, The University of Texas Rio Grande Valley, Edinburg, TX 78504 USA

**Keywords:** Agroecology, Behavioural ecology, Invasive species

## Abstract

In weeds, disturbance has been found to affect life history traits and mediate trophic interactions. In urban landscapes, mowing is an important disturbance, and we previously showed that continuous mowing leads to enhanced fitness and defense traits in *Solanum elaeagnifolium*, Silverleaf Nightshade (SLN). However, most studies have been focused on foliar defenses, ignoring floral defenses. In this study we examined whether continuous mowing affected floral defenses in SLN using mowed and unmowed populations in South Texas, their native range. We found flowers of mowed SLN plants larger but lighter than unmowed plants. Additionally, flowers on plants that were mowed frequently were both heavier and larger. Mowed plants had higher spine density and consequently unmowed flowers had higher herbivore damage. Additionally, early instar *Manduca sexta* fed on mowed flower-based artificial diets showed no difference in mass than the control and unmowed; however, later instars caterpillars on unmowed diets gained significantly more mass than the mowed treatment and control. Mowed plants had higher spine density which may shed light on why unmowed flowers experienced higher herbivore damage. We found caterpillars fed on high mowing frequency diets were heavier than those on low mowing frequency diets. Collectively, we show that mowing compromises floral traits and enhances plant defenses against herbivores and should be accounted for in management.

## Introduction

Weeds are thought of as undesirable plants in almost every ecosystem and environment and are usually examined through the lens of eradication or efforts to minimize their impact^1^. In agroecosystems, weeds are even more scrutinized as they harm both biotic and abiotic ecosystem services because of their enhanced traits allowing them to flourish in native and/or introduced habitats^2–4^. Enhanced weed traits are numerous and include the ability to outcompete heterospecifics, increasing fitness and defenses, as well as more vigorous germination rates. For example, in France Fried et al.^5^ found that the most ecologically successful weed species in maize crops were those with the C4 photosynthetic pathway and summer emergence. These weeds exhibited rapid resource acquisition through high specific leaf area (SLA) and high Ellenberg- (N) alongside, immense colonization capacity in the form of fecundity, seed longevity, and germination^5^. In agroecosystems worldwide, weeds have been shown to selectively produce seeds either pre- or post-crop harvest to avoid seed destruction alongside the harvest and for facilitation in seed dissemination^6^. Clearly, these traits make them of great interest toward identifying and understanding their success traits, with equal or more interest in how to diminish, manage and negate their impact.

While thousands of studies have demonstrated the effectiveness of chemical treatments on weeds,^7^ more recent studies have elaborated on these techniques and advanced toward specific practices, that can also be sustainable. For example, methods that target enzymes of plant-specific pathways to avoid toxicological side effects in non-target organisms, including mammals^8^. Sustainable approaches to weed management have gained tremendous interest and support, stemming from concerns including, but not limited to herbicide resistance in weed biotypes, a major concern in weed management^9^. For example, in the two Mediterranean weeds, *Diplotaxis erucoides* and *Erucaria hispanica*, a group^10^ found bipyridilium resistant biotypes with resistance to acetolactate synthase inhibitors, which alongside thousands of more studies highlight the difficulties in managing herbicide resistant weeds^11^. Regardless of the methodology or mode of action, understanding the factors and mechanisms underlying weed success is of primary importance in their management.

However, most methods of weed control seldom consider human-driven disturbances- such as extensive management practices. Weeds in urban and agriculturally managed systems have been documented to have increased success because of land and soil disturbances resulting from human- environmental interactions^12–14^. Clearing and draining promote erosion and damage non-weedy vegetation; however, this effect is not pronounced on weeds^15^. In addition, one of the most common human-disturbance through management practices throughout the world is mowing, occurring in commercial, urban, and agricultural landscapes. Since it is prevalent universally, mowing as a disturbance can yield valuable information that can possibly cause cascading effects on weed traits.

We had been documenting the impacts of mowing on weed traits, and our recent study^4^ demonstrated that *S. elaeagnifolium* (SLN) populations have higher fitness and foliar defense traits under continuous mowing. When comparing unmowed and mowed SLN populations in their native range, we found that while unmowed genets- non clonal individual plants, produced more fruits, seeds from mowed genets were significantly heavier, and similar to Fried et al.^5^, germination played a bigger role, and in mowed plants, the rate was higher. SLN is extremely rapid in establishing itself within a region, armed with multiple dispersal strategies and through its extensive taproot system which allows it to regenerate asexually and creates a significantly difficult management challenge^16^. Similarly, a study on the weed *Crepis sancta* showed increased dispersal and germination in urban patches^17^. Enhanced anti-herbivore defenses have also been shown to be a major component of weed success. Weeds have been found to show extraordinary adaptations to disturbance (e.g., common lambsquarters, field pennycress, giant foxtail, kochia) by increasing their chemical defenses when detecting disturbances in their seed banks in urban environments, and by increasing their physical seed defenses in undisturbed seed banks^18^. Extensive studies conducted on *Solanum carolinense* have described substantial constitutive and herbivore-induced physical and chemical defenses in the form of spines and trichomes, volatile and non-volatile compounds as a part of the foliar defense phenotype^19–23^. Similarly, a study found defense responses in Arabidopsis to foliar attacks via chemical means of jasmonic acid (JA) signaling^24^. Although these studies highlight a large swath of information toward weed fitness and defense as a result of disturbance; most of these studies are limited to foliar defenses and ignore floral traits affecting growth, and defenses. For example, a study^25^ examined the effects of herbivory on *Erythranthe guttata*and identified flower length as a key trait in estimating growth and fitness. In understanding weed success, seed fitness is a trait of prime importance in their ability to rapidly colonize and outcompete other vegetation and crops^4,26^.

We combined two relatively unexplored areas of weed biology to investigate the effects of an anthropogenic disturbance, mowing, on floral growth and defense traits, using a combination of field studies and lab experiments with genets from 6 mowed and 6 unmowed sub populations of SLN in its native range in south Texas. We asked the following questions: (1) Does continuous mowing influence floral grow and defense traits? (2) How does mowing affect field herbivory on floral organs (petals and anthers)? (3) How does a diet that contains the flower content from mowed and unmowed plants affect the growth and development of a voracious herbivore? (4) Do floral traits and herbivore performance additionally vary with the mowing frequency? Our research questions are based on the hypothesis that continuous mowing would result in reduced floral growth traits in mowed plants compared to unmowed ones. However, as a potential overcompensation, similar to plants’ local adaptation to mechanical wounding^4^, these mowed plants are expected to display more pronounced defense traits and have a greater impact on herbivores. To answer the herbivory question, we utilized the tobacco hornworm (*Manduca sexta*) caterpillar, a specialist on Solanaceae, previously observed on SLN in its native range in south Texas^27^ and known to be affected by the enhanced foliage defense following mowing. We also hypothesized that a higher frequency of mowing would also compromise growth and floral defense traits based on the defense-growth tradeoffs^22^.

## Materials and methods

### Study populations and plant materials

For all the experiments SLN, plant and flower material was collected from both mowed and unmowed areas from fields that are relatively close, spatially being within a 8 mile radius of each other, in the Rio Grande Valley (Mission, McAllen, Edinburg; Texas) in south Texas. SLN is native to southwestern United States and Mexico^28^, and we had been monitoring these populations over 4 years. Being native to the region, the obervation and collection required no permits or licensing. Throughout this monitoring, we have established that these SLN fields possess similar plant heights, levels of insect damage, numbers of insect herbivores, and types of insects feeding^29^.The mowed populations are on a fixed mowing schedule while the unmowed populations are undisturbed^4,14,27,29^. Voucher specimens for the species have been previously deposited (after identification) at UTRGV herbarium from the previous study that also led to this manuscript^4^.

### Flower collection

**Figure 1:**
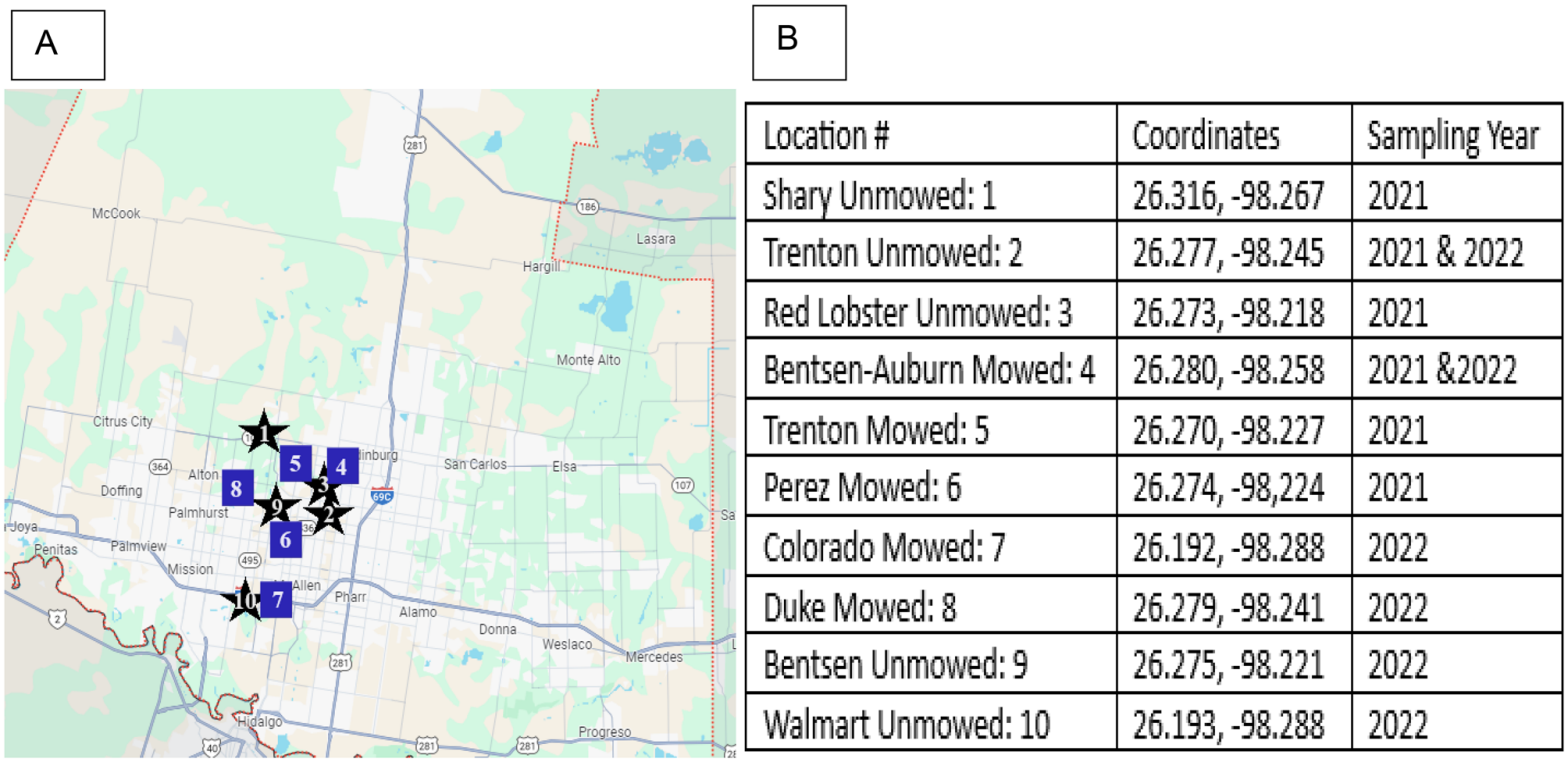
Geographic map of field collection sites (**A**) of *Solanum elaeagnifolium* flowers in Hidalgo County, Texas, USA, and their coordinates (**B**). Unmowed and mowed areas were annotated by squares or stars respectively. Map was obtained from Google Maps, 2024, *Map of Hidalgo County* (Google Maps, Google, Mountain View, California, USA). The locations were added to the map using Microsoft Paint 11.2309 (Microsoft, Redmond, Washington, USA).

In December 2021, flowers were collected from 6 populations (3 mowed and 3 unmowed). From mowed fields, 253 flowers were collected from 77 genets. From unmowed fields, 234 flowers were collected from 71 genets. Capable of vegetative propagation through rhizomes^28^ plants at least 3–5 m apart were considered as genets for sampling^30^ to minimize the risk of sampling clones. Flowers were all collected during the same week (all sampling within 3 days) and during the same time of day (8:00–9:00 A.M.) and stored in plastic bags until they were left to dry under ambient laboratory conditions (25 °C) for 48 h to remove excess water. In June 2022, flowers were collected again from 6 populations (3 mowed populations and 3 unmowed populations) from the fields previously mentioned (Fig. 1B). From mowed fields, 180 flowers were collected from 54 genets, and from unmowed fields, a total of 172 flowers were collected from 50 genets. Unequal sample sizes were a product of being unable to collect flowers from spatially close plants as the rhizomic reproduction of SLN requires it to ensure different genets. Flowers were all collected during the same week (all sampling done within 3–4 days) and during the same time of day (8:00–9:00A.M.) and stored in plastic bags until they were left to dry in ambient laboratory conditions (25 °C) for 48 h to remove excess water. The first round of collections was “high frequency mowing” as they were observed to be mowed for ~ 5 times between July and December. The second collection was considered “low frequency mowing” since they were the first set of flowers produced and were before the first mowing cycle (see Chavana et al.^4^ for more details).

### Floral traits measured

#### Floral diameter

All flowers were laid on a flat surface (anthers facing upwards to prevent damage to them) and their petal diameter (longest point) values were measured evenly and recorded in centimeters.

#### Floral mass

All flowers were weighed after 48 h of drying to obtain dry mass. For December 2021 flowers, anthers were removed and then weighed, as were petals for separate weights of each part.

#### Floral anther damage

All flowers were examined and assessed for damage on their anthers . Anther damage was assessed on a binary system, 0 for no damage and 1 for damage. Anther damage was categorized as damage on filament or anther sections of the stamen.

#### Floral petal damage

All flowers were examined and assessed for damage on their petals Petal damage was assessed on an ordinal scale of 0–3;0 being no damage, 1 being damage is present but not major, 2 being moderate damage, and 3 being severe damage as per the methods of Kariyat and Chavana^29^. All the data were collected by same individual to remove any subjective bias in deciding on the damage scale.

### Manduca sexta mass gain experiments

#### *Manduca sexta* colony

*Manduca sexta* eggs were purchased from a commercial vendor (Great Lakes Hornworm Ltd. Romeo, Michigan, USA) and maintained in the laboratory for 48 h before being used in this study. Eggs were randomly selected from different egg clusters for each of the following experiments.

#### Artificial diet for *Manduca sexta*

Artificial diets were prepared for rearing of *M. sexta* caterpillars. The artificial diet consists of a wheat-based germ diet prepared to specifications by suppliers (General Purpose Lepidoptera Diet (Product Code: F9772, Frontier Agricultural Sciences, Newark, DE, USA). The completed mixture was added to plastic Sterilite 6-Quart Storage Boxes (Walmart; Bentonville, AR, USA) and cooled down for 4 h at room temperature before refrigeration to serve as a control against artificial diet with different plant material mixtures. Please see Tayal et al.^31^ for more details about diet preparation. For 2021 floral collections, these diets were then modified by the addition of floral parts as mentioned in the following “High Mowing Frequency” section. For 2022 floral collections, these diets were modified by the addition of floral parts as mentioned in the “Low Mowing Frequency” section.

#### High mowing frequency mass gain diet experiment (2021 flower collections)

Alongside control diet, 12 additional diets were prepared using the plant material collected previously. Diets were prepared following the above specifications; however, each diet had added plant material relating to a population. Of the 12 diets prepared, 6 diets were made from unmowed plant material, and 6 diets from mowed plant material. Of the 6 diets made from unmowed plant material, 3 were prepared from anthers and 3 were prepared from petals, and these methods were followed for mowed plant material diets as well. Anthers were removed from flowers carefully, weighed, and crushed using a mortar for 30 min to create a very fine powder. This fine powder was added to the artificial diet mixture once cool to prevent the fine powder from being broken down by heat, mixed thoroughly, and stored. The same procedure was followed for petal plant material diets (90% weight germ wheat diet, 10% plant material based on our previous studies)^27,32^.

#### Low mowing frequency mass gain diet experiment (2022 flower collections)

Only 7 diets were prepared for the June 2022 Diet Experiment (3 mowed, 3 unmowed, 1 control) and ground plant material consisted of both anthers and petals for all treatments excluding the control treatment, as to have caterpillars fed on a diet absent mowed or unmowed plant parts for comparisons. Again, the same procedure was followed for plant material diets (90% weight germ wheat diet, 10% plant material based on our previous studies)^27,32^.

#### Experimental procedure and data collection

*Manduca sexta* eggs were separated into plastic containers labeled with each diet type (12 diets, 1 control diet, N = 390, for the 2021 High frequency mowing experiment; 6 diets, 1 control diet, N = 210 for the 2022 low frequency mowing experiment). 30 caterpillar eggs were placed in 2 plastic containers Sterilite 6-Quart Storage Boxes (Walmart; Bentonville, AR, USA) and fashioned with cardboard cuttings into cubicles, and labeled by caterpillar number (1–30) for data recording. Mass data was recorded every day at approximately the same time, morning, for 14 days for every caterpillar on each diet type. Plastic containers were lined with double-stacked paper towels cleaned every 2 days, and each caterpillar egg was placed on a fresh block of diet (1 cm^3^). Diet was taken out of the refrigerator 24 h before use to diminish cold shock effects and replaced every 2 days.

#### Spine density

Additional flowers were collected from the same 3 mowed and 3 unmowed fields sampled in June 2022. Flowers were collected randomly, but with a uniform collection method in which they were all cut from their plant at the first branching area to control for stem length. Flowers were taken back to the lab and spines were counted visually via hand-tally counter from the pedicel of the flower to the end of the stem This length was then measured to generate data based on number of spines, and spines per unit of length.

#### Polyphenol oxidase (PPO) assay

To test whether the flowers from mowed and unmowed plants differed in chemical defenses, we also measured their polyphenol oxidase activity- commonly used as a proxy for chemical defenses^27,33^. We quantified PPO content (U/mg) in SLN for the low frequency of mowing populations (3 mowed, 3 unmowed) using flower tissue samples (n = 8 per plant) from 3 separate genets from each field. The PPO assay performed as described in the Polyphenol Oxidase Assay Kit manual (Catalog#MBS822343; MyBioSource) with accordance with Watts and Kariyat 2022^32^. Quantification of PPO was performed using the equation in the Polyphenol Oxidase Assay Kit

$$ \begin{aligned} PPO \, \left( {U/g} \right) \, = & \, \left( {ODSample \, - \, ODControl} \right) \, \times \, VTotal \, / \, \left( {W \, \times \, VSample \, / \, VAssay} \right) \, / \, 0.01 \, / \, T \, \\ = & \, 233.3 \, \times \, \left( {ODSample \, - \, ODControl} \right) \, / \, W \\ \end{aligned} $$

where OD stands for calorimetric readout of optical density at 410 nm, VTotal is the volume of sample (0.35 ml), W is weight of the sample (0.1 g of plant tissue), VSample is the volume of sample (0.05 ml), VAssay is the volume of Assay buffer (1 ml) and T is the reaction time (3 min).

### Statistical analyses

Populations were pooled for the floral trait data. For each floral trait, the effect of the 15 mowing treatment (mowed vs. unmowed) or its frequency (low vs. high) was analyzed. The spine 16 density was analyzed by the mowing effect alone. For the floral traits, Analysis of Variance 17 (ANOVA) or T-test was used for continuous response values, whereas Ordinal Logistic 18 Regression was applied to discrete scale data. For caterpillar mass gain from artificial diets, mean 19 and daily masses of early (day 1–5) and late (day 6–13) instar caterpillars were analyzed using 20 Welch’s ANOVA to accommodate unequal variances or T-test where appropriate. All pairwise 21 post hoc comparisons were carried out using Tukey’s test. Mann–Whitney’s nonparametric test 22 was used to compare the Polyphenol Oxdiase (PPO) between unmowed and mowed treatments. 23 Full statistical models and details are presented in Table 1.

**Table 1:** Statistical details for the mowing effect and frequency, on floral traits of *Solanum elaeagnifolium*, mass gain of *Manduca sexta* caterpillars on artificial diets, and spine density on *Solanum elaeagnifolium* plants.

Trait	Source of variation	df	SS	F	P
Floral mass	Treatment (mowed/unmowed)	1	0.01645	67.1497	** < 0.0001**
	Frequency (high/low)	1	0.001146	4.6777	**0.0308**
Floral diameter	Treatment (mowed/unmowed)	1	1.58445	10.5446	**0.0012**
	Frequency (high/low)	1	12.7806	85.0557	** < 0.0001**
Early instar mass day 1–5	Treatment (mowed/unmowed/control)	2	N/A	2.24247	0.0894
	Frequency (high/low)	1	8195.8495	513.6042	** < 0.0001**
Late instar mass day 6–13	Treatment (mowed/unmowed/control)	2	N/A	3.7890	**0.0230**
	Frequency (high/low)	1	114,936,976	381.2343	** < 0.0001**
Spine density	Treatment (mowed/unmowed)	1	2708.3071	64.5082	** < 0.0001**
			**L-R ChiSquare**		
Petal damage	Treatment (mowed/unmowed)	1	14.44474		** < 0.0001**
	Frequency (high/low)	1	1.158853		0.2817
Anther damage	Treatment (mowed/unmowed)	1	1.589976		0.0273
	Frequency (high/low)	1	0.04125		0.8390

*P* values < 0.05 are in boldface.

## Results

### Floral traits

#### Floral diameter

**Figure 2:**
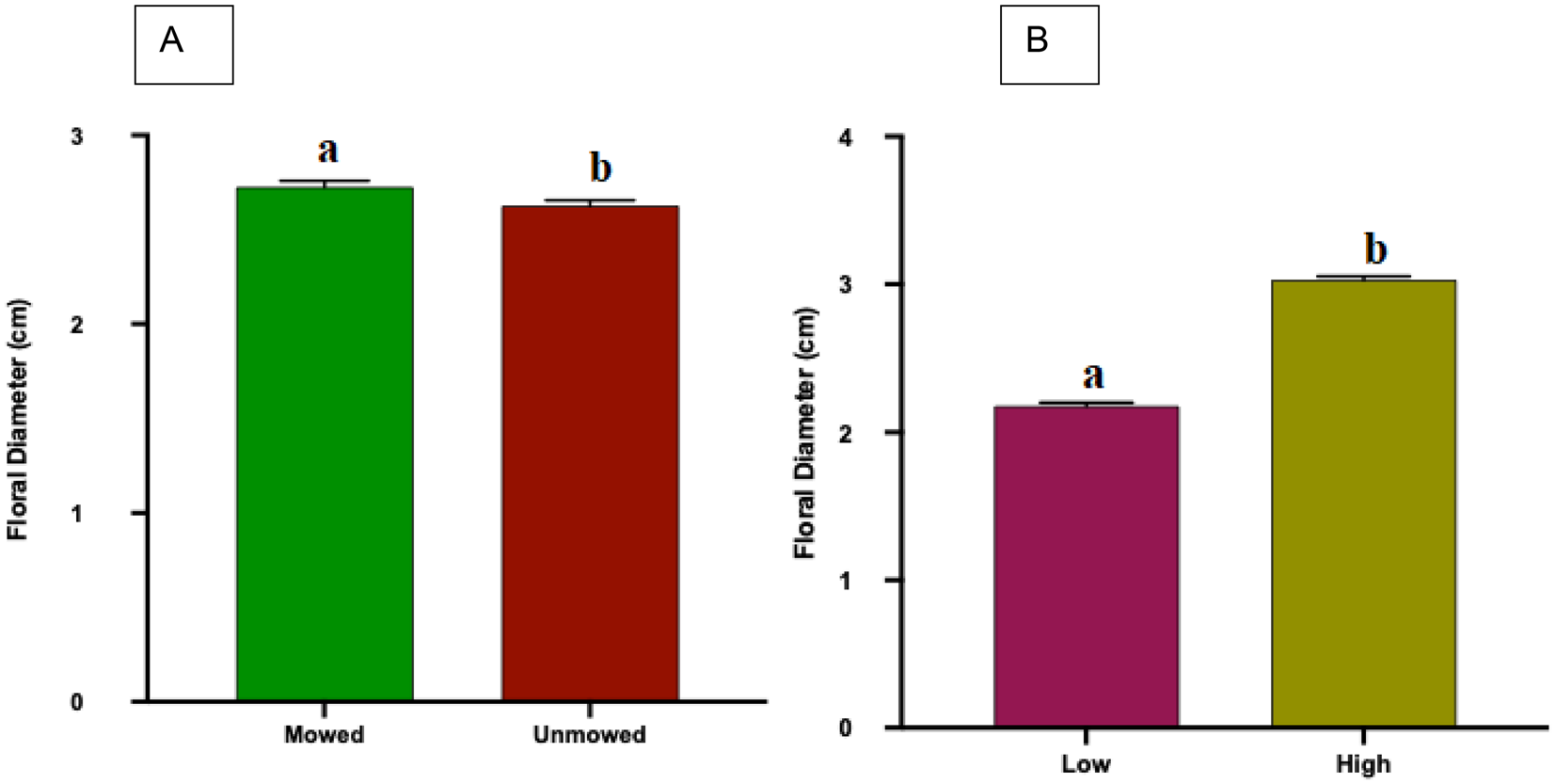
Flower diameter (mean + SE) of field collected *Solanum elaeagnifolium* plants in response to mowing treatment (**A**; unmowed and mowed) or the mowing frequency (**B**; low and high). Different letters above bars indicate significant difference (T-test, *P* < 0.05).

Analyses of floral diameter between mowed (Mean[cm] + / − SE, 2.726 ± 0.033) and unmowed (2.627 ± 0.0283) flowers showed that mowed plants had significantly larger flowers when compared to flowers from unmowed plants (T-Test: *P* = 0.0012, Fig. 2A). More interestingly, analyses of floral diameter between low (2.179 ± 0.021) and high (3.039 ± 0.026) mowing frequency plants showed significantly larger flowers from high mowing frequency plants when compared to flowers from low mowing frequency (T-Test: *P* < 0.0001, Fig. 2B).

#### Floral mass

**Figure 3:**
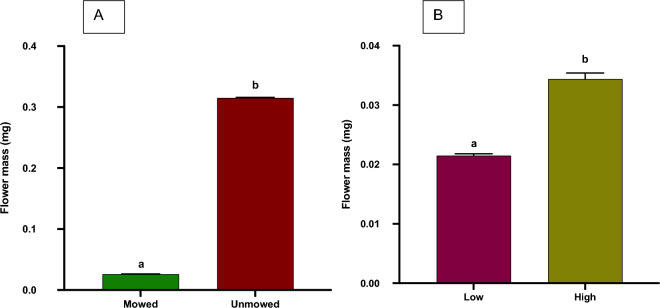
Flower mass (mean + SE) of field collected *Solanum elaeagnifolium* plants in response to the mowing treatment (**A**; unmowed and mowed) or the mowing frequency (**B**; low and high). Different letters above bars indicate significant differences (T-test, *P* < 0.05).

Analyses of floral mass between mowed (Mean[mg] + / − SE, 0.0266 ± 0.0004) and unmowed (0.0316 ± 0.0012) flowers show significantly heavier flowers from unmowed plants when compared to flowers from mowed plants (T-Test: *P* < 0.0001, Fig. 3A). Analyses of floral mass between low (0.0215 ± 0.0003) and high (0.0344 ± 0.001) mowing frequency plants showed that significantly heavier flowers were from high mowing frequency plants when compared to flowers from low mowing frequency (T-Test: *P* = 0.0308, Fig. 3B).

#### Anther damage

Anthers from the flowers of unmowed plants and mowed plants had no significant difference in damage incidence (Logistic Regression; *P* = 0.2073). Regarding mowing frequency, there was also no significant difference between low and high frequency of mowing flowers (Logistic Regression; *P* = 0.8390).

#### Petal damage

**Figure 4:**
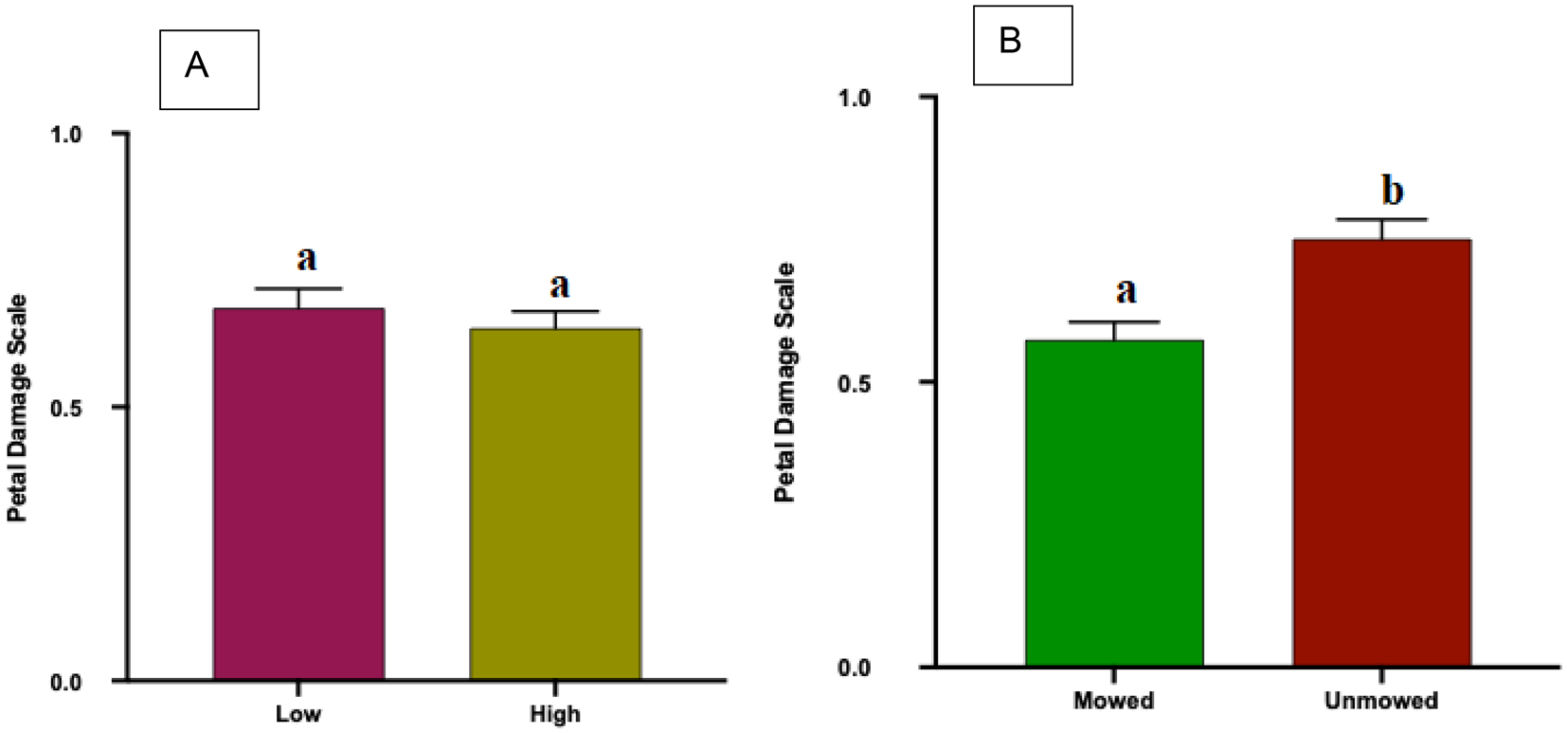
Petal damage scores (0–3 scale, mean + SE) of field collected *Solanum elaeagnifolium* plants obtained from the mowing treatment (**A**; unmowed and mowed) or the mowing frequency (**B**; low and high). Different letters above bars indicate significant difference ( Ordinal Logistic Regression, *P* < 0.05).

Petals from the flowers of unmowed plants had significantly more damage (0–3 scale) on than mowed plants (Ordinal Logistic Regression: *P* < 0.0001, Fig. 4A). However, there was no significant difference in petal damage between petals of high mowing frequency flowers and low mowing frequency flowers (Ordinal Logistic Regression: *P* = 0.2817, Fig. 4B).

### Manduca sexta mass diet experiments

#### Early instar mass

**Figure 5:**
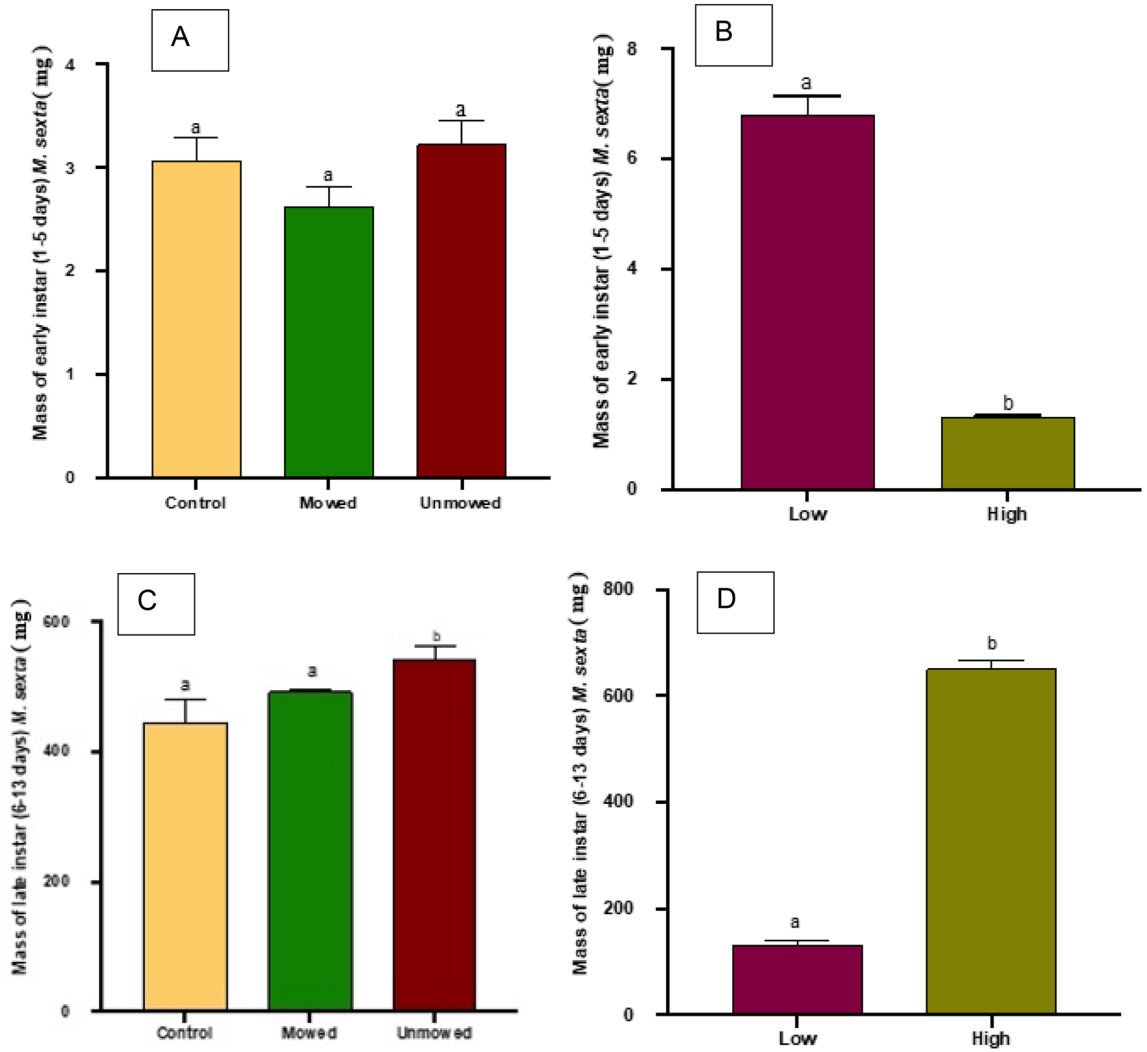
Mean masses (+ SE) of early (1–5 days after hatching; **A**, **B**) and late (6–13 days after 3 hatching; **C**, **D**) instar Manduca sexta caterpillars fed on artificial diets created from flowers of 4 *Solanum elaeagnifolium* plants following the mowing treatment (**A** and **C**) or different mowing 5 frequencies (**B** and **D**). Different letters indicate significant differences among the control, mowed, and unmowed treatments (Tukey’s tests on Welch’s ANOVA, *P* < 0.05), or between the low and high frequency of mowing (T-Test, *P* < 0.05).

Analyses of *M. sexta* mass in early instars (Day 1–5) showed no significant differences between caterpillars fed on unmowed (Mean[mg] + / − SE, 3.230 ± 0.224), mowed (2.631 ± 0.183), and control (3.074 ± 0.226) (Welch’s Test for Unequal Variance: *P* = 0.0894, Fig. 5A). With regard to frequency, early instar *M. sexta* fed on low (6.780 ± 0.355) frequency of mowing diets were significantly heavier than those fed on high frequency of mowing diets (1.308 ± 0.0418) (T-Test: *P* < 0.0001, Fig. 5B).

#### Late instar mass

Analyses of *M. sexta* mass in late instars (Day 6–13) show significant differences between caterpillars fed on unmowed (543.8 ± 18.23), mowed (492.6 ± 20.48), and control (444.5 ± 35.30) diets with caterpillars fed on unmowed diets being significantly heavier than both mowed and control caterpillars (Welch’s Test for Unequal Variance: *P* = 0.0230, Fig. 5C). With regard to frequency, late instar *M. sexta* fed on high (650.6 ± 15.84) frequency of mowing diets were significantly heavier than those fed on low mowing frequency diets (132.3 ± 6.905) (T-Test: *P* < 0.0001, Fig. 5D).

#### Effect of anthers and petals in diet on daily mass

**Figure 6:**
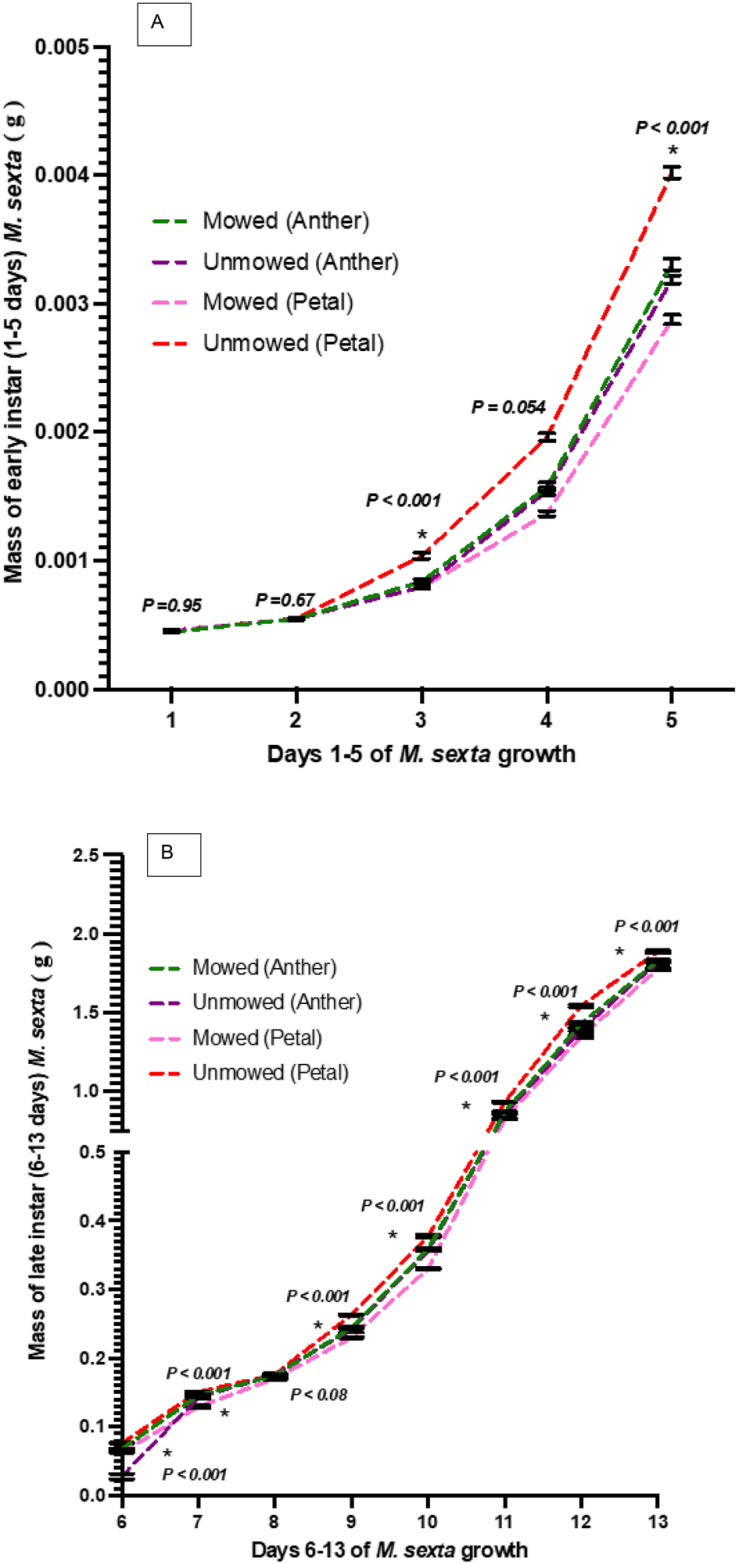
Daily mass analysis for (**A**) early instar (1–5 days) and (**B**) late instar *Manduca sexta* caterpillars on artificial diets containing anther and petal powders from *Solanum elaeagnifolium* plants without mowing (unmowed) or with high-frequency mowing (mowed) management. The four treatments were compared by Welch’s ANOVA (*P* < 0.05).

Analyses of *M. sexta* mass with regard to diets prepared in the High Mowing Frequency Period (December 2021) show that between diets made from petals and anther SLN floral parts, caterpillars gain significantly more mass on petal diets on days 3, 5, 6, 11, and 12 (ANOVA: *P* < 0.0001, *P* < 0.0001, *P* < 0.0001, *P* = 0.0018, *P* = 0.0003, Fig. 6A,B). However, caterpillars on days 4, 7, 8, and 10 gained significantly more mass on anther diets (ANOVA: *P* < 0.0001, *P* = 0.0028, *P* = 0.0095, *P* = 0.0055, Fig. 6A,B). Clearly, floral parts added diet had differential effects on early and late instar growth of the specialist herbivore.

### Spine density

#### Number of spines per unit length

**Figure 7:**
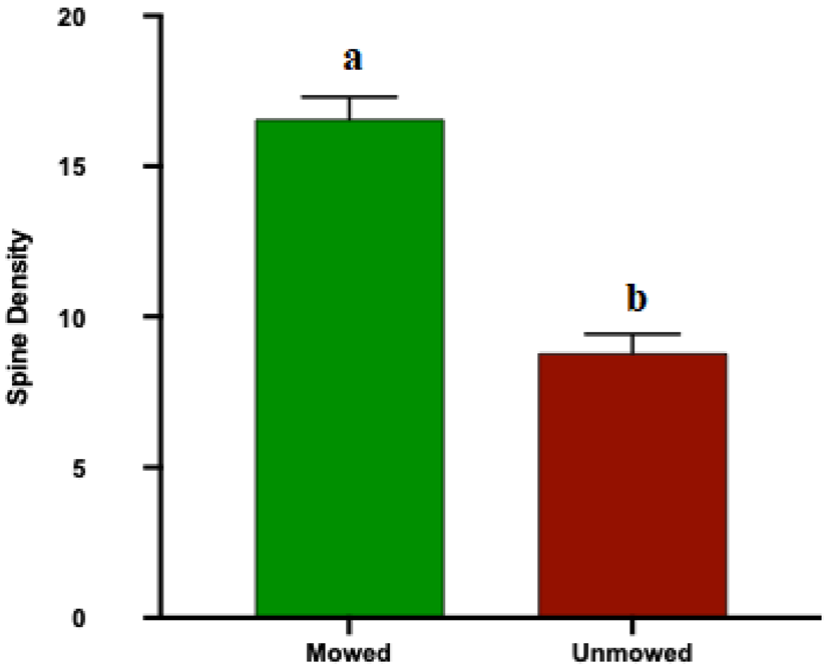
Mean (+ SE) spine density of *Solanum elaeagnifolium* flower pedicels from mowed and unmowed treatments. Different letters indicate significant differences between mowed and unmowed treatments determined by post hoc analyses using T-Test (*P* < 0.05).

Analyses of number of spines per unit length of mowed (Mean [Number of spines/1 cm] + / − SE, 16.5693 ± 0.67189) and unmowed (8.8072 ± 0.69468) flowers show that mowed flowers have significantly more spines per unit length than unmowed flowers (ANOVA: *P* < 0.0001, Fig. 7).

### Polyphenol oxidase (PPO)

**Figure 8:**
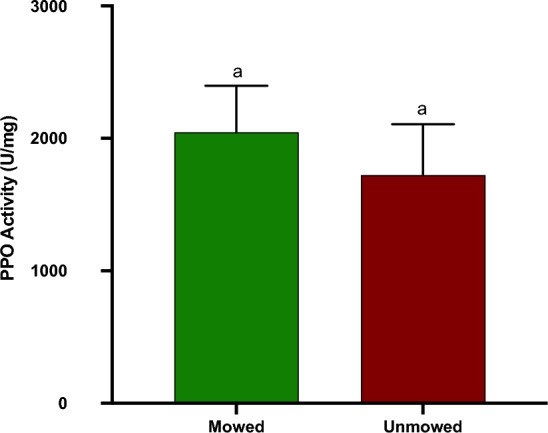
Mean (+ SE) Polyphenol activity of flowers from mowed and unmowed *Solanum elaeagnifolium* plants. Mann–Whitney analysis shows that the treatments did not statistically differ at α = 0.05.

Analyses of Polyphenol Oxidase shows that PPO activity between mowed and unmowed flowers from low mowing frequency SLN was not significantly different (Mann Whitney: *P* = 0.5039, Fig. 8).

## Discussion

In this study, we examined how disturbance (mowing) and frequency of mowing affects floral traits, plant defenses, and herbivory on SLN. Interestingly, we found that mowed flowers had larger diameters than unmowed flowers, but flower mass was in line with our hypothesis as unmowed flowers were heavier in high mowing frequency populations. Polyphenol Oxidase (PPO) content was found to not be significantly different between mowed and unmowed treatments, which indicates perhaps lower concentration of the plant defense compound in floral parts as opposed to leaves where it is known to deter herbivores^34,35^. We also found that mowed flowers had significantly less damage on petals, indicating that the stress due to continuous mowing potentially led to an increase in the induction of defense traits, although there was no effect of frequency on petal and anther damage. To compound these effects, we also show that mowed flowers had higher spine density, a major anti-herbivore defense in Solanaceae^21,36^. Taken together, we show that mowing increased floral defense traits, and for a species like SLN that produce over ~ 100 flowers per plant, has tremendous consequences for spread, dispersal, and invasion success^28,37^.

While examining the effects of floral traits modified by mowing, we incorporated floral organs into artificial diet, and found interesting results. In early *M. sexta* instars, we found no significant difference between larvae fed on control, unmowed, and mowed diets (although mowed diet-fed individuals were smaller than all treatments.). In late instars, there was no significant difference in mass between larvae fed on control and mowed diets; however, unmowed diet fed caterpillars were significantly heavier than their control and mowed counterparts, which may indicate that the effect of mowing as a disturbance is more pronounced on late instar *M. sexta*, which corresponds to trends we have noticed in a fewstudies^23,38,39^. This again also echoes the findings of petal and anther damage: that continuous mowing as a stress positively impacts and contributes to floral defenses. While data analyses of mass of *M. sexta* between anther and petal fed caterpillars was inconclusive, when pairing anthers and petals with mowed and unmowed treatments, we see that caterpillars fed on both unmowed and petal diets gained significantly more mass than the other 3 treatments (unmowed x anther, mowed x anther, and mowed x petal) which continues to support unmowed plants having less herbivore defenses as we have found before^4^. Collectively, these results are also supported by previous findings that has shown that food quality effects are more pronounced in early instars of holometabolous insects when manipulated^40^.

Consequently, our data supports the possibility that increased defenses as a result of disturbance have contributed to SLN mitigating herbivore damage and outcompeting non-weedy plants in South Texas (Fig. 1A). These results coincide with our previous work on SLN which found similar results with regard to SLN defense against herbivores as a result of mowing, showing *M. sexta* gaining significantly less mass on mowed plants^4^. Additionally, we reiterate the importance of showing germination rates from Chavana et al. 2021, in which mowed SLN plants had increases germination rates as a result of continuous mowing because it enhances the ability of SLN to not only diminish herbivore stress but also benefit from anthropogenic stress, and that the methods meant to control these weeds actually leads to them returning more rapidly to the environment. Following this, SLN growing in urban soils have been observed to have reduced herbivory as well, supporting their ability to thrive in disturbed, urban environments where other plants cannot^14^.

With regard to mowing frequency, which has less previous data to draw conclusions upon, early instar mass was larger within low mowing frequency individuals and the opposite effect was found in late instar larvae as high mowing frequency individuals were significantly heavier. A group^41^ found detrimental effects because of repeat disturbances on multiple invertebrate taxa including insects, which aligns itself with the lower mass of high mowing frequency larvae observed during the early instars. This, however, contradicts the late instar masses as they were higher in high frequency of mowing, yet this can potentially be attributed to stress-induced vigorous growth responses^42^. One consideration is potential differences between the high and low mowing frequency collections, as they were collected in different years; however, we found no differences between mowed or unmowed low and high frequency experiments. Although we could not disentangle the effects of mowing frequency, our findings show that mowing as a disturbance has some contribution to detrimental effects on the Solanaceae specialist herbivore, *M. sexta*.

Taken together, our data supports the premise that mowing has significant impact on floral traits, affecting both their growth traits but also their ability to defend themselves against herbivore insects. Mowing as a disturbance has strong indications of being an important environmental anthropogenic disturbance that may be damaging to urban agricultural areas and therefore needs to be better understood. For example, while our data from field and lab showed defense trait induction, our experiments did not have enough resolution to examine spatial and temporal variation in defenses, including secondary metabolites, and gene expression^20^. While could potentially expect to see a reduction in herbivores in mowed fields, we did examine more complex trophic consequences for mowing, including the attraction of pollinators and predators^43^. And finally, additional experiments should also examine how mowing affects floral scent with possible consequences for pollination, as most buzz pollinating species^43^, use multi modal host selection (flower size, color, flower density, and scent^44^ in Solanum genus.

### Data collection

The authors confirm that the experimental research and field studies on plants (either cultivated or wild), including the collection of plant material, follows relevant institutional, national, and international guidelines and legislation.

### Data sharing

The datasets used and/or analyzed during the current study available from the corresponding author (rkariyat@uark.edu) on reasonable request.

## References

[1] Stefan, L., Engbersen, N. & Schöb, C. Weeds are not always evil: Crop-weed relationships are context-dependent and cannot fully explain the positive effects of intercropping on yield. *bioRxiv* (2020). 10.1101/2020.04.02.021402v1. 10.1002/eap.2311 33630392

[2] Yaisys, B. Review the role of weeds as a component of biodiversity in Agroecosystems. *INCA* (2016). Available at: https://www.researchgate.net/publication/308730965_Review_The_role_of_weeds_as_a_component_of_biodiversity_in_agroecosystems. (Accessed: 4th November 2022)

[3] Neve P (2018). Reviewing research priorities in weed ecology, evolution and management: A horizon scan. Weed Res..

[4] Chavana J, Singh S, Vazquez A, Christoffersen B, Racelis A, Kariyat RR (2021). Local adaptation to continuous mowing makes the noxious weed *Solanum elaeagnifolium* a superweed candidate by improving fitness and defense traits. Sci. Rep..

[5] Fried G, Chauvel B, Munoz F, Reboud X (2019). Which traits make weeds more successful in maize crops? Insights from a three-decade monitoring in France. Plants.

[6] Clements DR, Jones VL (2021). Ten ways that weed evolution defies human management efforts amidst a changing climate. Agronomy.

[7] Imran, Amanullah (2022). Assessment of chemical and manual weed control approaches for effective weed suppression and maize productivity enhancement under maize-wheat cropping system. Gesunde Pflanzen.

[8] Kraehmer H, Schulz A, Rosinger C, Laber B (2014). Herbicides as weed control agents: State of the art: I. Weed control research and safener technology: The path to modern agriculture. Plant Physiol..

[9] Monteiro A, Santos S (2022). Sustainable approach to weed management: The role of precision weed management. Agronomy.

[10] Travlos I, de Prado R, Chachalis D, Bilalis DJ (2020). Herbicide resistance in weeds: Early detection, mechanisms, dispersal, new insights and management issues. Front. Ecol. Evolut..

[11] Peleg Z, Lati R (2021). Herbicide resistance in weed management. Agronomy.

[12] Lozon JD, MacIsaac HJ (1997). Biological invasions: Are they dependent on disturbance?. Environ. Rev..

[13] DiTomaso JM (2000). Invasive weeds in rangelands: Species, impacts, and management. Weed Sci..

[14] Kasper S, Chavana J, Sasidharan L, Racelis A, Kariyat R (2021). Exploring the role of soil types on defense and fitness traits of silverleaf nightshade (*Solanum elaeagnifolium*), a worldwide invasive species through a field survey in the native range. Plant Signal. Behav..

[15] Goslee SC, Peters DPC, Beck KG (2001). Modeling invasive weeds in grasslands: The role of allelopathy in acroptilon repens invasion. Ecol. Modell..

[16] Roberts J, Florentine S (2022). A review of the biology, distribution patterns and management of the invasive species *Amaranthus palmeri* S. Watson (*Palmer amaranth*): Current and future management challenges. Weed Res..

[17] Cheptou PO, Carrue O, Rouifed S (2008). A Cantarel The streak-end rule: How past experiences shape decisions – pnas.org. Biol. Sci..

[18] Davis, A. S., Schutte, B. J., Iannuzzi, J. & Renner, K. A. Chemical and physical defense of weed seeds in relation to soil seedbank persistence: Weed science. *Weed Science* (2017). Available at: https://www.cambridge.org/core/journals/weed-science/article/chemical-and-physical-defense-of-weed-seeds-in-relation-to-soil-seedbank-persistence/3B6742ADEA4894657EB841C36F5B65D0. (Accessed: 3rd November 2022)

[19] Kariyat RR, Mauck KE, De Moraes CM, Stephenson AG, Mescher MC (2012). Inbreeding alters volatile signalling phenotypes and influences tri-trophic interactions in horsenettle (*Solanum carolinense* L.). Ecol. Lett..

[20] Kariyat RR (2012). Inbreeding, herbivory, and the transcriptome of solanum carolinense. Entomologia Exp. et Applicata.

[21] Kariyat RR, Balogh CM, Moraski RP, De Moraes CM, Mescher MC, Stephenson AG (2013). Constitutive and herbivore-induced structural defenses are compromised by inbreeding in solanum carolinense (Solanaceae). Am. J. Bot..

[22] Kariyat RR, Sinclair JP, Golenberg EM (2013). Following Darwin's trail: Interactions affecting the evolution of plant mating systems. Am. J. Bot..

[23] Kariyat RR, Portman SL (2016). Plant-herbivore interactions: Thinking beyond larval growth and mortality. Am. J. Bot..

[24] Ballaré CL, Austin AT (2019). Recalculating growth and defense strategies under competition: Key roles of photoreceptors and jasmonates. J. Exp. Bot..

[25] Ivey CT, Carr DE (2005). Effects of herbivory and inbreeding on the pollinators and mating system of *Mimulus guttatus* (Phrymaceae). Am. J. Bot..

[26] Bigio, L., Lebel, M. & Sapir, Y. Do different measures of maternal fitness affect estimation of natural selection on floral traits? A lesson from Linum pubescens (linaceae). *Journal of Plant Ecology* (2016). Available at: https://academic.oup.com/jpe/article/10/2/406/2624147. (Accessed: 3rd November 2022)

[27] Watts S, Kariyat RR (2021). Picking sides: Feeding on the abaxial leaf surface is costly for caterpillars. Planta.

[28] Petanidou T (2018). Pollination and reproduction of an invasive plant inside and outside its ancestral range. Acta Oecologica.

[29] Kariyat RR, Chavana J (2018). Field data on plant growth and insect damage on the noxious weed *Solanum eleaegnifolium* in an unexplored native range. Data Brief.

[30] Kariyat RR, Scanlon SR, Mescher MC, De Moraes CM, Stephenson AG (2011). Inbreeding depression in solanum carolinense (Solanaceae) under field conditions and implications for mating system evolution. PLoS One.

[31] Tayal M, Somavat P, Rodriguez I, Martinez L, Kariyat R (2020). Cascading effects of polyphenol-rich purple corn pericarp extract on pupal, adult, and offspring of tobacco hornworm (*Manduca sexta* L.). Commun. Integrat. Biol..

[32] Watts S, Kariyat R (2022). Are epicuticular waxes a surface defense comparable to trichomes? A test using two solanum species and a specialist herbivore. Botany.

[33] War AR (2012). Mechanisms of plant defense against insect herbivores. Plant Signal. Behav..

[34] Ludlum CT, Felton GW, Duffey SS (1991). Plant defenses: Chlorogenic acid and polyphenol oxidase enhance toxicity of *Bacillus thuringiensis* subsp.kurstaki to Heliothis zea. J. Chem. Ecol..

[35] Singh S, Kariyat RR (2020). Exposure to polyphenol-rich purple corn pericarp extract restricts fall armyworm (spodoptera frugiperda) growth. Plant Signal. Behav..

[36] Kariyat RR, Hardison SB, De Moraes CM, Mescher MC (2017). Plant spines deter herbivory by restricting caterpillar movement. Biol. Lett..

[37] Krigas N, Tsiafouli MA, Katsoulis G, Votsi NE, van Kleunen M (2021). Investigating the invasion pattern of the alien plant *Solanum elaeagnifolium* Cav. (silverleaf nightshade): Environmental and human-induced drivers. Plants.

[38] Kaur J, Kariyat R (2020). Role of trichomes in plant stress biology. Evolut. Ecol. Plant-Herbivore Interact..

[39] Tayal M (2020). Polyphenol-rich purple corn pericarp extract adversely impacts herbivore growth and development. Insects.

[40] Holmes LA, Nelson WA, Lougheed SC (2020). Food quality effects on instar-specific life histories of a holometabolous insect. Ecol. Evolut..

[41] Haghkerdar JM, McLachlan JR, Ireland A, Greig HS (2019). Repeat disturbances have cumulative impacts on stream communities. Ecol. Evolut..

[42] Price PW (1991). The plant vigor hypothesis and herbivore attack. Oikos.

[43] Tayal M, Kariyat R (2021). Examining the role of buzzing time and acoustics on pollen extraction of *Solanum elaeagnifolium*. Plants.

[44] Kariyat RR, Bentley TG, Nihranz CT, Stephenson AG, De Moraes CM, Mescher MC (2021). Inbreeding in solanum carolinense alters floral attractants and rewards and adversely affects pollinator visitation. Am. J. Bot..

